# Experiments on the Livers of Birds, in Relation to the Presence of Sugar

**Published:** 1852-12

**Authors:** 


*Experiments on the Livers of Birds, in relation to the presence of Sugar.*
By George D. Gibb, M. D., L. R. C. S. I., Hon. Fellow Med. Soc. Va.; Lecturer on the Institutes of Medicine, St. Lawrence School of Medicine, Montreal; Physician to the Montreal Dispensary.

During my residence in France, in 1848, M. Claude Bernard published a paper in the Archives Générales de Médecine, upon the source of sugar in the animal economy. I was presented with a copy of this paper through the politeness of the author.*

His experiments on the liver, demonstrating the existence of sugar as a natural constituent of that organ, were principally confined to the dog species, and I have repeatedly confirmed them in the human subject, and in many other animals of the class Mammalia.

The healthy liver of man and animals is now proved to contain sugar as a normal constituent; but in certain diseases, particulary those of a tuberculous character, as a pulmonary phthisis for example, where we sometimes find the liver enlarged, and in the condition termed "fatty" by Louis, the amount of sugar present appears to be very great indeed.

To determine whether this rule, the natural existence of sugar in the livers of Mammalia, would stand good with respect to another class of the Vertebrata, namely, birds, which rank next in importance to Mammals, I instituted a series of experiments.

It will be remembered, that, in birds, the liver is a viscus of considerable magnitude, consisting of two principal lobes, and firmly suspended in situ by ligaments and membranous processes. The vena porta, supplying that venous blood from which the bile is elaborated, is formed by vessels, derived from numerous sources, receiving not only the veins of the stomach, spleen, and intestines, as in Mammalia, but likewise the renal and sacral veins.†

There is also a difference in the amount of fat contained in the livers of the different orders of birds. Thus in the Palmipedes or webfooted birds, and the Grallæ or Waders, the larger number of species possess quantities of fat in their livers. In the Gallinæ or Poultry, again, in very many species there is a notable absence of fat.

The quantity present, or absent, of the fat, influences the amount of sugar to be detected, at least such is the conclusion numberless experiments lead me to.

If, again, the hepatic cells of the livers of birds are examined with the microscope, they are found even more free from fat globules than are those of Mammalia, and they are almost entirely filled with amorphous biliary particles.‡

In the following experiments, the livers were pounded in a clean mortar to a pulp, then boiled in a very small quantity of water for some minutes, and filtered. After cooling, the filtered fluids were examined. They are examples selected from a number:

---

* An abridged translation is published in 5th volume *British American Medical and Physical Journal.*

† Rymer Jones' Comparative Anatomy.

‡ Principles of Physiology, General and Comparative, by W. B. Carpenter, edition 1851.

No. 1. *Small Chicken.*

*Moore's Test* gave the merest trace of sugar.

*Trommer's* shewed its presence satisfactorily, but still in small quantity.

*Cappezuoli's* was also satisfactory, the yellow deposit of oxide of copper being pretty clear, after the lapse of some hours.

No. 2. *Larger chicken* than the last was killed by dividing the jugular vein, collecting the blood as it flowed. This fluid was allowed to separate into its two portions, and the serum examined for sugar, but no satisfactory results were obtained.

The liver was treated in the usual way.

*Moore's Test* shewed the presence of sugar greater than in the last experiment, but still in small quantity.

*Trommer's* was pretty satisfactory, and shewed the presence of a tolerable quantity.

*Cappezuoli's* was also equivocal, more so than in the last experiment.

No. 3. Liver of a *Fowl.*

*Moore's Test* light brown.

*Trommer's*, very marked indeed.

No. 4. Liver of *another Fowl.*

*Moore's Test* pale brown.

*Trommer's*, darker brown.

No. 5. Liver of a *Turkey.*

*Moore's Test* light brown.

*Trommer's*, darker brown.

No. 6. Liver of a *Goose.*

Both Tests very much marked indeed, indicating a large quantity of sugar.

No. 7. Liver of a *Duck.*

Both Tests marked in a similar degree to the goose.

Sugar was detected in every bird's liver I examined, the quantity being in proportion to the amount of fat present, and this was invariably large in the webfooted or water birds. There is a striking analogy to this in the Phocida, among the mammalia animals living almost entirely in the water, as the Walrus and Seal, and in which their livers are found to be almost masses of fat, and the quantity of sugar in that of the Seal is enormous.

M. Bernard, in his experiments, examined the blood as well as the liver, and found sugar to be a normal ingredient in both. I was unable to examine the blood, excepting in one instance, and discovered none.

To pursue these investigations further, experimental examination should be made on the livers of reptiles and fishes, which are store-houses of fat and oil; the livers of cod and other large fishes prove this from their yielding a considerable supply of the latter. And the great bulk of the liver in the Crustacea, Mollusca, and cold-blooded Vertebrata just mentioned, has reference apparently, not to a large production of bile, but to an accumulation of fat.

The deductions to be drawn from the fact of sugar existing in the liver and blood, cannot as yet be satisfactorily arrived at, until our knowledge is farther advanced on the subject. M. Bernard considers that a regular function of the liver is the formation of sugar, and that the liver alone has the power of producing sugar without starch. The sugar, as it is formed, is conveyed away by the hepatic veins, the vena cava inferior and right side of the heart; and as none is found in the pulmonary veins returning from the lungs,

Magendie infers, that it must have undergone destruction in the lungs and the carbon eliminated.*

The presence of sugar in the blood of the portal vein, which takes venous blood from the intestines and other viscera to the liver, is accounted for by M. Bernard, by the regurgitation of the blood from the liver, when the pressure of the abdominal parietes is removed on opening the abdomen; and this is permitted, he says, by the absence of valves. In this view, I cannot altogether coincide with the author, but do believe that the sugar found in the vena porta is totally independent of that in the liver itself, probably arising in most instances from the mesenteric veins.—*Canada Med. Journal.*

* See Dunglison's Human Physiology, for a clear consideration of these experiments, vol. ii, 1850, a work that ought to be in the library of every inquiring physician.

